# Hearing Screening among First-Grade Children in Rural Areas and Small Towns in Małopolskie Voivodeship, Poland

**DOI:** 10.3390/audiolres11020025

**Published:** 2021-06-15

**Authors:** Weronika Swierniak, Piotr Henryk Skarzynski, Elzbieta Gos, Natalia Czajka, Monika Matusiak, Patryk Hartwich, Magdalena Beata Skarzynska

**Affiliations:** 1 Department of Teleaudiology and Screening, World Hearing Center, Institute of Physiology and Pathology of Hearing, 10 Mochnackiego Street, 02-042 Warsaw, Poland; w.swierniak@ifps.org.pl (W.S.); e.gos@ifps.org.pl (E.G.); n.czajka@ifps.org.pl (N.C.); 2 Heart Failure and Cardiac Rehabilitation Department, Faculty of Medicine, Medical University of Warsaw, 8 Kondratowicza Street, 03-242 Warsaw, Poland; 3 Institute of Sensory Organs, 1 Mokra Street, 05-830 Nadarzyn/Kajetany, Poland; m.skarzynska@csim.pl; 4 Department of Oto-Rhino-Laryngosurgery, World Hearing Center, Institute of Physiology and Pathology of Hearing, 10 Mochnackiego Street, 02-042 Warsaw, Poland; m.matusiak@ifps.org.pl; 5 Otolaryngology Clinical Department, Collegium Medicum, The Jagiellonian University, 2 Jakubowskiego Street, 30-688 Cracow, Poland; phartwich@gmail.com; 6 Centre of Hearing and Speech Medincus, 05-830 Kajetany, Poland

**Keywords:** hearing screening, hearing loss, prevalence, school screening, pure tone audiometry

## Abstract

Undiagnosed hearing deficits hamper a child’s ability to learn. Hearing screening in school aged children helps detect educationally significant hearing loss and prevents negative impacts on academic achievement. The main purpose of this study was to improve early detection and assess the incidence of hearing disorders in first-graders from rural areas and small towns in the Małopolskie Voivodeship of Poland. There were 5029 children aged 6–7 years. Hearing thresholds were measured over the frequency range 0.5–8 kHz. A result was considered positive (abnormal) if the hearing threshold was worse than 20 dB HL at one or more frequencies. The prevalence of hearing loss was estimated in terms of four-frequency hearing loss, high-frequency hearing loss, and low-frequency hearing loss. Parents filled in a brief audiological questionnaire. The analysis was performed using IBM SPSS Statistics, version 24. Of all the children, 20.5% returned a positive result and were referred for further audiological diagnoses. The estimated prevalence of hearing loss was 11.6%, made up of 6.5% with FFHL, 7.6% with HFHL, and 8.2% with LFHL. This study showed that large numbers of children in the district had hearing problems. Adoption of hearing screening in primary schools is recommended as a routine procedure within preventive pediatric health care.

## 1. Introduction

Hearing loss has been classified as one of the most common causes of disorders among conditions arising in chronic disease and trauma. This dysfunction has been ranked higher than many other chronic diseases such as diabetes, dementia, and chronic obstructive pulmonary disease [1]. The ability to hear is essential, particularly during children’s education in primary school. Listening skills are important when learning to read and write, and have a relevant impact on the development of social skills [2]. Hearing disorders are amenable to intervention; therefore, a screening program has value when the child is beginning school. School-based hearing screening programs provide an opportunity to minimize the inequities between children caused by hearing loss. However, even in countries where routine newborn screening is conducted, childhood hearing loss is an under-recognized public health problem [3,4,5]. Newborn screening does not detect hearing disorders that occur later in childhood, such as drug-related ototoxicity, infection, and otitis media with effusion [6,7,8]. One of the most common disorders in childhood, especially in low and middle-income countries and rural areas is otitis media [9,10,11,12,13]. Approximately 80% of school aged children have had an episode of temporary hearing loss due to otitis media [13]. However, temporary hearing disorders, such as conductive hearing loss, are often overlooked by parents or caregivers. Thus, hearing screening programs in schools may help to identify children with hearing disorders that occur after birth [14]. That solution could help to fill the gap in preventative care occurring after the period of newborn hearing screening through access to the whole population of children in one place while utilizing the integrated educational infrastructure and school nurses [15].

The European Consensus on hearing, vision, and speech screenings in preschool and school aged children encouraged the implementation of the preschool hearing screening program [7,16]. However, despite the recommendations of professional organizations, currently there is no known standardized hearing screening program for school aged children in Poland, there are only local programs. Furthermore, as opposed to newborn hearing screening, the protocols used in school hearing screening programs vary widely [17,18,19].

In 2008, the Institute of Physiology and Pathology of Hearing in collaboration with the Polish Agricultural Social Insurance Fund and the Association of the Deaf and Hearing Impaired ‘Homo-Homini’ performed the first stage of screening examinations for children attending rural schools and small towns (below 5000 inhabitants) in eastern Poland, during which 92,876 pupils were examined [20]. Afterwards, in the same partnership, the program was extended to western Poland [18]. This study presents the results of the next stage of the program in the Małopolskie Voivodeship.

The main goal of the program was to increase early detection and assess the number of hearing disorders in first grade primary school children from rural areas and small towns from the Małopolskie Voivodeship. The second goal was to increase the knowledge in the participants of the program about the potential causes of hearing disorders and the possibilities of prevention, diagnosis, treatment, and rehabilitation when it occurs. Information materials about the program, along with a prevention booklet and consent forms for the examination, were distributed to schools through local government units (municipalities). The teachers distributed the materials among parents during regular meetings. Cooperation between regional councils and local authorities with the school helped to increase the school attendance in the program.

## 2. Materials and Methods

### 2.1. Participants

Hearing screening was performed in 630 primary schools located in 19 communities in the Małopolskie Voivodeship. Significant difficulties in program implementation were the high dispersion of schools in the regions covered by the program. The rural regions were predominantly small schools with less than twenty children participating in the program. The initial study sample consisted of 5038 students. Nine children with a prior clinical diagnosis of any hearing impairment were excluded from the analysis. Finally, there were 5029 children, including 2015 children aged 6 years and 3014 children aged 7 years. The study sample consisted of 2281 girls and 2748 boys.

### 2.2. Measurements

The examination was performed using the Sensory Organs Examination Platform, which was developed by the Institute of Physiology and Pathology of Hearing and the Institute of Sensory Organs [21,22]. The platform is equipped with Sennheiser HDA200 headphones, which provide effective acoustic insulation for the ear against environmental noise. The platform allows the user to conduct screening based on pure tone audiometry. The feature allows the technician to perform an air conduction audiometric test for each ear separately over the frequency range 0.5–8 kHz and for levels not exceeding 80 dB HL. Screening pure tone audiometry was conducted by certified technicians in quiet classrooms in accordance with the modified Hughson and Westlake procedure [17,23]. Only the air conduction threshold was measured. Hearing thresholds were determined for the right and left ear of each patient at frequencies of 0.5–8 kHz. According to previously established criteria, an audiometric test was considered abnormal (positive), if the hearing threshold was above 20 dB HL at one or more frequencies in at least one ear [21,24]. The results of the audiometric hearing tests were automatically collected in the “SZOK”^®^ central database, through which it is possible to perform a statistical analysis and transfer collected data to the Institute Physiology and Pathology, where they can be evaluated by a specialist audiologist or ENT. The collected results were marked with a unique identifier, which guarantees full protection of personal data of persons under examination in accordance with applicable law.

Audiometric hearing tests were supplemented by the results of a questionnaire filled in by parents or legal caregivers. The questionnaire included five questions: Do you think your child has any problems with his/her hearing? Does your child complain of tinnitus in their ears/head when in quiet? Does your child often listen to loud music? Has your child been treated for otitis media? Does your child complain about noise at school? The response format was yes or no.

### 2.3. Data Analysis

A result of the screening was regarded as positive (refer) if a hearing threshold was above 20 dB at one or more frequencies in at least one ear. Hearing loss was defined as a pure-tone average higher than 20 dB in at least one of the following pure-tone averages: four-frequency pure-tone average (FFPTA), high-frequency pure-tone average (HFPTA), and low-frequency pure-tone average (LFPTA). Firstly, speech relevant hearing loss was defined as the four-frequency pure-tone average (FFPTA) at 0.5, 1, 2, and 4 kHz at a value for a threshold of >20 dB [25,26]. HFPTA were defined as a pure-tone average above 2 kHz (4 and 8 kHz) [22,27] and LFPTA at 0.5, 1, 2 kHz [24,28] at a value for a threshold of >20 dB HL. Unilateral hearing loss was diagnosed when there was normal hearing in one ear and hearing loss in the other [26].

The prevalence of hearing loss in children was estimated by dividing the number of cases by the total number of individuals. Additionally, 95% confidence intervals (95% CIs) were calculated to indicate uncertainty of estimates. A chi-square test for independence was conducted to assess the relationship between the child’s hearing status and the parental questionnaire. Statistical significance was established as a *p*-value of <0.05. The analysis was performed using IBM SPSS Statistics, version 24.

## 3. Results

### 3.1. Positive Results of Hearing Screening

Positive results of the hearing screening were obtained in 1032 out of 5029 children (i.e., 1032 children had an elevated threshold for at least one frequency). This rate was 20.5% (95% CI, 19.4–21.6%). There were 20.6% of the girls (95% CI, 19.0–22.3%) and 20.4% of the boys (95% CI, 18.9–21.9%) with a positive outcome.

### 3.2. Prevalence of Hearing Loss

There were 581 children with hearing loss, i.e., having FFPTA HL, and/or LFPTA HL, and/or HFPTA HL in at least one ear. The estimated prevalence of HL was 11.6% (95% CI, 10.7–12.4%). The prevalence of the three types of hearing loss was estimated in all children, and in girls and boys separately, and is shown in Table 1.

Unilateral hearing loss was found in 388 children, 7.7% (95% CI, 7.0–8.5%), and bilateral hearing loss was found in 193 children, 3.8% (95% CI, 3.3–4.4%).

### 3.3. Questionnaire Results

The parents’ answers were compared to their children’s hearing status (normal hearing vs. hearing loss). It was found that parents of children with hearing loss more often suspected problems with hearing in their children (12.5%; *n* = 66) in comparison to parents of the children with normal hearing (5.6%; *n* = 227). The relationship was statistically significant, χ^2^ = 36.74; *p* < 0.001.

**Figure 1:**
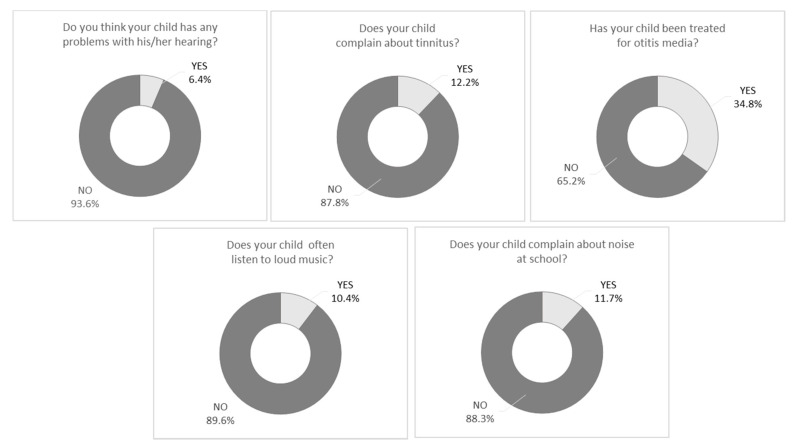
Results of the questionnaire.

Our study also showed that, in the parents’ opinion, children with hearing loss more often listened to loud music (13.8%; *n* = 73) than children with normal hearing (9.9%; *n* = 401), χ^2^ = 7.59; *p* = 0.006. We did not find any statistically significant relationship between the children’s hearing status and tinnitus (χ^2^ = 1.55; *p* = 0.213), otitis media (χ^2^ = 0.03; *p* = 0.954), or complaining about school noise (χ^2^ = 0.04; *p* = 0.948). The questionnaire was completed by 4564 parents. Their answers are shown in Figure 1.

## 4. Discussion

Our study revealed that 20.5% (1032 children) had a positive result. All children with a positive result were referred for a further diagnosis to an audiologist or ENT specialist. In comparison, the prevalence of hearing loss children attending rural schools and small towns throughout Poland has been estimated as 16.4% [22]. Data from pilot hearing screening projects, conducted by the Institute of Physiology and Pathology of Hearing in various African countries, have shown the prevalence of hearing loss (18–34%) [29], and in Asian countries the prevalence is 15.9–24.1% [21,26]. For comparison, the percentage of children in India with hearing loss was found to be 11.9% (more than 1 in 8 children) [30], and in Iran it was found that 10% of children aged 7–8 years old may have a hearing problem [31]. Govender and Mars [32] assessed 146 ears of learners at a school from South Africa and found that 23 ears of 20 children (16%) presented with hearing loss. The prevalence of four-frequency hearing loss in the present study was 6.5%, slightly higher than the 5.6% in the study by Skarzynski et al. [22]. Additionally, the results presented in this study are also higher than those (4.7%) obtained by Feder et al. [25]. Differences in prevalence may be due to different sample sizes, different assessment protocols [33], and by the various ages of the children. In addition, the prevalence of hearing loss in children in developed countries is typically lower than in developing countries [34]. Fortnum et al. [4] suggested that the lack of hearing screening programs, lack of education about hearing disorders, and limited access to health care in underserved areas are the reasons for these differences.

Low-frequency hearing losses were identified in 8.2% of the tested children in the Małopolskie Voivodeship. The data reported in another study conducted in Poland indicated the rate of low-frequency HL was estimated to be 6.2% [22]. For comparison, data from a study of an American population indicated a higher incidence of LFHL of 7.1% [35], and in Kyrgyzstan, results of the hearing screening indicated such an incidence in 7.2% of children [26]. In some cases, a low-frequency hearing loss may be temporary [36] and, depending on the specifics of the individual case, pharmacological or surgical intervention may be effective. The most common reasons for this kind of hearing loss are cerumen, perforation of the ear drum, tympanosclerosis, and otitis media with effusion [37]. These conditions make the child more tired in the classroom because of the increased effort needed to listen. The child may have difficulty with speech understanding in certain situations, such as understanding faint or distant speech, and can seem inattentive or distracted in the classroom [38].

High-frequency hearing losses were identified in 7.6% of the tested children in the Małopolskie Voivodeship. Our result is in line with results reported in the study conducted by Johnson et al. [27] on 2867 children in the United States which also found a 7.6% rate of HFHL. Causes of high-frequency hearing loss in children can be noise, diseases, ototoxicity, infections, or it may also be caused by genetic factors [39]. Children with HFHL may appear inattentive or distractible due to difficulties understanding speech in the noise. Recess can be very noisy, which can lead to social problems if a child is unable to hear or misinterprets information during those situations. In HFHL, speech disorders and articulation problems can arise. It is important that children with HFHL should be permanently supported in school and in their home environment [40].

Our findings showed that unilateral hearing loss (UHL) was more frequent (7.7%), than bilateral hearing loss (BHL) (3.8%), which is in line with results reported by a previous study in Poland [22]. In the USA, 3–6% of school aged children have some degree of UHL [41]. Binaural hearing offers the listener several important advantages over monophonic hearing. It has been established that binaural hearing provides better speech perception, better sound localization, increased loudness perception through binaural summation, and an overall improvement in hearing in both noisy and quiet environments [42].

Results of the hearing screening obtained due to audiometric testing were supplemented with information from the questionnaire completed by parents. We found that 6.4% of the parents suspected some problems with their child’s hearing. This figure was not high, and in fact it was lower than the estimated prevalence of hearing loss in the children. Moreover, the parents’ opinions were not accurate. Admittedly, in the group of hearing loss, parents suspected that there was a problem with their child’s hearing (12.5%) significantly more often than in the group with normal hearing (5.6%); however, parental perception was still not appropriate. These results are in line with other researchers’ findings [43,44,45] and has shown that parents have low levels of sensitivity to hearing problems in their children. However, the reason for this remains an open question. One hypothesis may be a lack of knowledge about hearing disorders and their associated consequences. It is also likely to be due to objective difficulties: identifying mild or single-sided hearing impairment is a challenging task for parents who have neither the expertise nor the precise instruments to detect hearing impairment. Psychological factors may also be important, for example, parents want to have a healthy child and therefore tend to underestimate any worrisome symptoms. Further research is needed to better understand this phenomenon. However, the practical conclusion of our study is that there is a great need to educate parents about the symptoms of hearing disorders and early detection.

Our study revealed that 12.2% of the parents indicated that their children have tinnitus. In the recent systematic review undertaken by Rosing et al. [46] it was stated that the estimated prevalence of tinnitus was from 4.7% to 46% in the general pediatric population, so the figures vary widely. Raj-Koziak et al. [47] emphasized that children rarely complain spontaneously of tinnitus and their parents are unaware of the condition. Tinnitus may be an independent symptom, but also it may precede full-blown hearing loss, so attention should be paid to tinnitus during routine pediatric check-ups.

Our screened children were first-graders, and they were 6–7 years old. Even so, 10.4% of their parents reported that the children often listen to loud music. Leisure time noise and loud music are a known risk factor for noise-induced hearing loss [48,49,50,51]. Our study confirms these reports: we found that significantly more children with hearing loss were exposed to loud music (13.8%) than children with normal hearing (9.9%). It should also be noticed that school noise is a real problem for first-graders. We found that 11.7% of the parents said that their children often complained about noise at school. As was demonstrated by Jamieson et al. [52] the youngest school aged children are highly susceptible to the noise in a classroom, which has a serious negative effect on their school performance. Our study shows a need to reduce noise levels in home, recreational, and school settings.

A study conducted by Harmes et al. [53] reported that an estimated 80% of the pediatric population have at least one episode of acute otitis media (AOM), and approximately 80% to 90% will have at least one incident of otitis media with effusion (OME) before reaching school age. In the current study, 34.8% of parents indicated that their child was treated for otitis media. Although most infections are a result of bacterial invasion, AOM is a consequence of Eustachian tube dysfunction that develops from an acute viral infection of the upper respiratory paths [54]. The middle ear inflammation is one of the most common reasons for temporary hearing loss [55]. During this period, preventive measures should be taken to maximize the hearing ability of affected children at home and at school. These include advising teachers to let children sit nearer the front of the classroom, and giving advice to their parents and educators to talk to their students face to face. Parents need to understand that because their child has fluid in the middle ear, they will not hear well, and behavioral problems may be due to frustration [56]. Therefore, an important task is to find the best ways to educate parents and teachers about the symptoms of hearing impairment in their children [17].

This study has revealed that hearing problems are common among first-grade students at primary schools in rural areas. There are many factors that prevent children from receiving medical care. Such obstacles involve a lack of available transportation to medical centers, the parents’ inability to both take time off from their jobs, and the family’s lack of financial resources. Thus, there is a need to consider whether participation in hearing screening for first-graders should be mandatory, or at least strongly recommended by central or local education authorities. The first step is to appoint a project coordinator at a a local authorites -level. It should be a local person who can collaborate with the community and coordinate information, resources, and program services. For large schools it would also be useful to establish a school coordinator (e.g., school nurse, speech pathologist, school counselor) who is able to get in touch with children and their parents.

Inexpensive hearing screening examinations could improve the hearing health of children from rural areas. With appropriate screening equipment and protocols, and in close collaboration with well-trained personnel at the school (for example, school nurses), it is feasible to conduct hearing screening among school aged children. The proposed protocol and the Sensory Organs Examination Platform used in the current study are cost-effective and safe tools and appear to be a successful method for identifying children with postnatal hearing disorders, allowing the provision of early intervention services in a timely manner.

### Limitations

This was a cross-sectional study; therefore, it was not feasible to distinguish between congenital and acquired HL to identify if any of the cases had developed or progressed to HL. A prospective study would be needed to establish this, but such a study would be difficult due to the technical and infrastructure difficulties. This project would require repeated hearing tests for toddlers, and preschool children to identify those children who acquire losses (Wake et al., 2006). Moreover, methods used in the program did not differentiate between permanent and periodic hearing disorders. In addition, future research should consider a teacher questionnaire regarding noticing the prevalence of hearing problems among students.

## 5. Conclusions

Routine hearing screening among school aged children is still not part of public health policy in Poland. Hearing screening is an important element of health education and should be aimed not only at children, but also at parents and teachers. There is still a need to improve early detection of hearing impairment in children starting their school education. It is recommended that conditions for screening school aged children be established in terms of study population, methodology, criteria used to determine which children should be referred, and the staff and equipment needed.

## Figures and Tables

**Table 1:** Prevalence of hearing loss in children aged 6–7 years of the Małopolskie Voivodeship.

	FFPTA and/or LFPTA and/or HFPTA HL				FFPTA HL			LFPTA HL			HFPTA HL		
	*N*	*n*	%	95% CI	*n*	%	95% CI	*n*	%	95% CI	*n*	%	95% CI
Total	5029	**581**	**11.6**	10.7–12.4	**328**	**6.5**	5.8–7.2	**410**	**8.2**	7.4–8.9	**384**	**7.6**	6.9–8.4
Girls	2281	**257**	**11.3**	10.0–12.6	**163**	**7.1**	6.1–8.2	**200**	**8.8**	7.6–9.9	**157**	**6.9**	5.8–7.9
Boys	2784	**324**	**11.8**	10.6–13.0	**165**	**6.0**	5.1–6.9	**210**	**7.6**	6.6–8.6	**227**	**8.3**	7.2–9.3

FFPTA, four-frequency pure-tone average; HFPTA, high-frequency pure-tone average; LFPTA, low-frequency pure-tone average; HL, hearing loss; N, study sample size; n, number of participants with positive result; CI, confidence interval.

## Data Availability

Data available upon request.
